# Adipose tissue: an overlooked target of medical ionizing radiation and its metabolic consequences

**DOI:** 10.3389/fpubh.2026.1953578

**Published:** 2026-09-14

**Authors:** Maria José Pereira, Daniela Rosendo-Silva, Inês A. Marques, Joana Santos, Paulo Matafome, Ana Margarida Abrantes

**Affiliations:** 1 Instituto Português Oncologia de Coimbra Francisco Gentil, E.P.E., Coimbra, Portugal; 2 ESTESC - Coimbra Health School, Polytechnic University of Coimbra, Coimbra, Portugal; 3 Faculty of Sciences and Technology, University of Coimbra, Coimbra, Portugal; 4 Faculty of Medicine, Coimbra Institute for Clinical and Biomedical Research (iCBR) and Institute of Physiology, University of Coimbra, Coimbra, Portugal; 5 Center for Innovative Biomedicine and Biotechnology (CIBB), University of Coimbra, Coimbra, Portugal; 6 Clinical Academic Center of Coimbra (CACC), Coimbra, Coimbra, Portugal; 7 Faculty of Medicine, Institute of Biophysics, Coimbra Institute for Clinical and Biomedical Research (iCBR) Area of Environment Genetics and Oncobiology (CIMAGO), University of Coimbra, Coimbra, Portugal; 8 ESTESC - Coimbra Health School, Health and Technology Research Center (H&TRC), Polytechnic University of Coimbra, Coimbra, Portugal

**Keywords:** adipocytes, ADSCs, ionizing radiation, metabolic health, radiation effects

## Abstract

Despite extensive research on radiation effects on various tissues, the impact on adipose tissue remains largely unexplored. This represents a critical knowledge gap given adipose tissue’s role as an active endocrine organ and the extensive use of ionizing radiation in medical procedures. This review synthesizes the current evidence regarding the effects of ionizing radiation on adipose tissue pathophysiology, examining molecular mechanisms and clinical implications for metabolic health. We comprehensively reviewed studies investigating radiation effects on adipocytes and adipose-derived stem cells (ADSCs), analyzing different radiation energies, dose rates, and exposure timeframes. Converging evidence demonstrates that different dose protocols with ionizing radiation induce heterogeneous effects on adipose tissue cell number and size, pro-inflammatory secretome, and ADSCs functionality. Clinical studies in childhood cancer survivors reveal strong associations between abdominal radiation exposure and subsequent metabolic dysregulation, including insulin resistance and diabetes. Mechanistic studies suggest radiation-induced oxidative stress, impaired adipogenesis, and altered adipokine secretion as key pathways. Overall, ionizing radiation disrupts adipose tissue function through multiple molecular pathways, potentially predisposing individuals to long-term endocrine and metabolic dysfunctions. Elucidating the underlying mechanisms will contribute to optimize radiation therapy protocols and guide the development of targeted strategies to preserve metabolic health.

## Introduction

Electromagnetic radiation can be classified into non-ionizing and ionizing categories based on their energetic characteristics. While non-ionizing radiation (infrared, visible light, microwaves, radio waves) lacks sufficient energy to remove electrons from atoms, ionizing radiation possesses enough energy to ionize atoms, with alpha and beta particles, gamma rays, and X-rays serving as prime examples (1–3). The discovery of X-rays by Wilhelm Conrad Roentgen made ionizing radiation indispensable in clinical practice, encompassing both diagnostic imaging and therapeutic applications, particularly radiotherapy. Indeed, the most of the exposure from human-made sources arises from medical approaches. This widespread medical utilization makes it imperative to understand radiation effects on healthy tissue, which may constitute a significant public health concern (3–9).

Ionizing radiation exposure triggers diverse biological effects resulting in complex responses at molecular, cellular, and tissue levels. These effects can be classified as direct or indirect effects. Direct effects refer to the ionization of atoms within critical biomolecules, such as deoxyribonucleic acid (DNA), lipids, and proteins, leading to molecular damage that may result in mutations or cell death. Indirect effects, which account for the majority of radiation-induced biological damage, are characterized predominantly by the radiolysis of water molecules and the formation of free radicals, including reactive oxygen species (ROS), capable of inducing cellular damage (3).

Radiation exposure disrupts normal cellular and extracellular matrix homeostasis, activating signaling pathways that upregulate inflammatory cytokines, trigger coagulation cascade activation, cause vascular damage, induce cell death, and lead to organ dysfunction. These effects manifest acutely through inflammatory mechanisms or chronically, depending on tissue structure, cellular composition, proliferation rate, metabolic activity, vascularisation, radiosensitivity, and dose-time parameters (3, 6, 7, 9–13).

The adipose tissue is extensively distributed throughout the human body and serves as its main energy reservoir while functioning as a sophisticated endocrine organ, comprising distinct cellular and functional compartments that may respond differently to radiation exposure. Although the available evidence supports the effects of ionizing radiation in white adipose tissue, brown adipose tissue, although recognized as an important regulator of systemic energy metabolism, has not been systematically investigated after ionizing radiation. White adipose tissue comprises two distinct compartments: subcutaneous adipose tissue (SAT), located between the body surface and the external muscular layers, and visceral adipose tissue (VAT), surrounding internal organs. VAT exhibits greater metabolic activity and stronger correlation with obesity-related health complications, demonstrating reduced insulin sensitivity, decreased vascularisation, elevated pro-inflammatory macrophages, increased leptin and resistin secretion, and enhanced lipolytic activity (14–17).

Structurally, white adipose tissue contains large lipid-filled adipocytes, extracellular matrix composed of collagen, reticular and elastic fibers, and the stromal vascular fraction (SVF). This fraction encompasses immune cells, endothelial cells, hematopoietic and mesenchymal stem cells, preadipocytes, fibroblasts, and adipose-derived stem cells (ADSCs) (9, 18, 19). ADSCs represent multipotent mesenchymal stem cells capable of adipogenic, osteogenic, and chondrogenic differentiation, playing crucial roles in tissue homeostasis and regeneration. As an endocrine organ, adipose tissue produces adipokines—hormones, growth factors, and cytokines secreted by both adipocytes and stromal vascular cells. These bioactive molecules regulate metabolic homeostasis, appetite, insulin secretion, blood pressure, coagulation pathways, and tumor development, underscoring adipose tissue’s systemic importance (9, 18–24).

Compelling epidemiological evidence links radiation exposure to long-term metabolic complications. Children surviving leukemia and lymphoma demonstrate significantly higher obesity and metabolic syndrome prevalence, with insulin resistance being particularly prominent (25). Although most patients undergo external beam radiotherapy, the underlying mechanisms have remained largely unexplored until recent investigations. Two pivotal clinical studies (26, 27) provided evidence linking radiation exposure during childhood with later metabolic dysregulation. First, Friedman et al. (26) revealed that cancer survivors under 21 years exposed to abdominal radiation face an increased risk for insulin resistance and diabetes, without evidence of beta-cell dysfunction, a finding consistent with adipose tissue involvement (26). Second, Huang et al. (27) demonstrated that adult survivors of childhood cancer, exposed to therapeutic abdominal radiation or total body irradiation, exhibit subcutaneous adipose tissue dysfunction characterized by macrophage activation and impaired adipokine secretion (27).

While the effects of radiation have been extensively studied in most tissues, its impact on adipose tissue remains largely unexplored and poorly understood. Adipose tissue is still largely perceived as a fat storage tissue, instead of the active endocrine tissue and the metabolic regulator it is. Disturbances in adipose tissue function may lead to severe peripheral metabolic consequences (Figure 1) due to the dysregulation of lipid and glucose metabolism and impaired adipokine secretion (18, 28). In fact, dysregulation of adipokine secretion has been extensively demonstrated as being related to cancer etiology. This may be related to the increased secretion of factors involved in establishing the proinflammatory milieu of the tissue and promoting cell proliferation (19). Radiation is known, for instance, to have an incidence on cardiac toxicity (heart failure, ischemic heart disease and stroke) (29). Thus, similar effects may occur in adipose tissue, with unpredictable consequences for the future metabolic health of the patients. This review aims to summarize the current state of the art regarding adipose tissue changes after exposure to ionizing irradiation and their metabolic consequences later in life.

**Figure 1:**
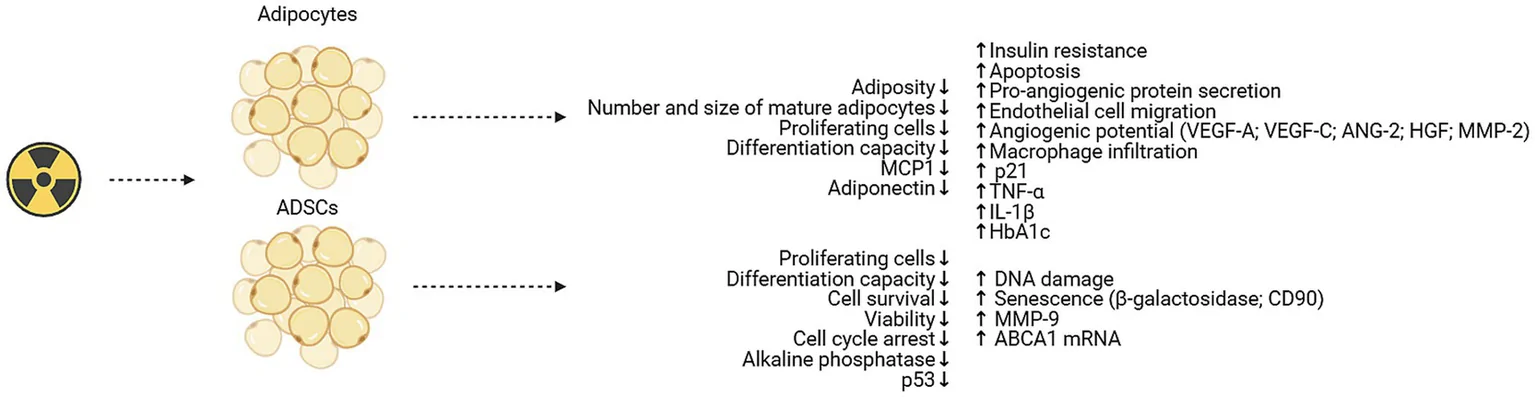
Overview of the most radiation-induced adipose tissue dysfunction. Schematic representation of the principal cellular and molecular responses triggered by ionizing radiation (created with BioRender.com).

## Radiation-induced adipose tissue dysfunction

The characterization of molecular pathways underlying radiation-induced adipose tissue dysfunction is essential for elucidating the origins of radiation-induced metabolic disorders. Therefore, this review provides current knowledge on adipose tissue responses to ionizing radiation and their potential metabolic implications. To provide an overview of the current state of the art, Table 1 summarizes the radiation protocols (dose and dose rate), radiation type, adipose tissue cell type, and the main effects on adipose tissue cells. Understanding dose–response relationships is crucial for clinical translation. Diagnostic procedures typically deliver doses below 0.1 gray (Gy), while therapeutic radiation ranges are usually from 20 to 80 Gy in fractionated protocols (30, 31). Dose evaluation is complex due to the different energy sources and dose ranges used in the investigations over the years. Considering the energy, the existing studies can be divided mostly into X-rays and gamma emission (Cs^137^ and Co^60^), classified as low linear energy transfer (LET) radiation types (3). Few studies estimate the biological effects of low and high LET in the same study, which is very important due to the different amounts of transferred energy per unit length. Besides that, the energy imparted to a material, represented by Gy, also diverges, from very low doses such as 0.01Gy to much higher doses such as 30 Gy. In most studies, the dose of ionizing radiation delivered per time unit (dose rate) was too low (0.2 Gy/min) or too high (7.31 Gy/min). This high variability of energy absorption rate makes the analysis of the biological effects more difficult (3). Only one study (21) distinguished biological results from low LET as X-ray and high LET from carbon ion. Besides that, only two studies divided the results from different dose classifications as lethal (10 Gy) and sublethal (7 Gy) doses (9) or low (0.1–0.3 Gy) and higher (0.5–1 Gy) doses (6). Even relatively low doses demonstrate measurable effects on adipose tissue function, raising concerns about cumulative exposure effects and individual radiosensitivity variations (9, 32).

**Table 1:** Radiation protocols summary of studies reviewed and main ionizing radiation effects on adipose tissue cells, respectively.

References	Radiation protocol	Radiation type	Cell type	Analyze type	Main conclusion
Adipocytes					
Marques et al. (6)	Low doses (0.1; 0.3 Gy) Higher doses (0.5; 0.8; 1Gy)	X-rays	Murine 3 T3-L1 pre-adipocyte (differentiated adipocytes status at irradiation)	*In vitro*	- ↑ pro-angiogenic protein secretion - ↑ endothelial cell migration - ↑ angiogenic potential *in vitro*
Poglio et al. (9)	10Gy (lethal); 7Gy (sublethal) 4.4Gy/min (total body irradiation)	γ-rays (Cs^137^)	C57BL/6 mice (5–6 weeks old)	*In vivo* *In vitro* (SVF cells analysis)	- ↓ fat pad weight - ↓ mature adipocyte number and size - ↓ proliferating cells (Ki-67) - ↑ apoptotic cells (TUNEL) - ↓ resident hematopoietic cells in adipose tissue - ↓ SVF progenitor function
Ablamunits et al. (43)	8Gy (total body irradiation)	γ-rays (Cs^137^)	B6.V-*Lep*^ob/ob^ and B6.V-*Lep*^+/?^ mice with bone marrow transplantation (leptin-deficient, genetically obese with 2 months old)	*In vivo*	- Arrest body weight gain (2 months post-irradiation) - Hepatomegaly - ↓ MCP-1 expression - ↑ insulin resistance - ↓ adiposity
Okada et al. (44)	850 cGy (abdominal region irradiation)	γ-rays	B6.V-*Lep*^ob/ob^ and B6.V-*Lep*^+/?^ mice (9 weeks old)	*In vivo*	- ↓ adiposity - Induced expression of p21, TNF- α, and IL-1β - ↑ hepatic lipid content - ↑ expression of lipogenic genes - Slightly ↑ macrophage infiltration
Ruggiero et al. (35)	Mice model:^1^ 7Gy in 2 fractions of 3.5Gy 0.358 Gy/min Nonhuman primate model:^2^ 6MV 4Gy 0.8 Gy/min (total body irradiation)	^1^γ-rays (Co^60^) ^2^X-rays	- 8-week-old C57BL/6 mice - rhesus macaques	*In vivo*	- ↓ CD34^+^ adipose progenitors - ↓ differentiation capacity - ↓ adipose progenitor proliferation and differentiation
Bacarella et al. (42)	6MV 4Gy 0.8Gy/min (total body irradiation)	X-rays	Nonhuman primates: rhesus macaques	*In vivo*	- ↓ adipose accumulation - ↑ HbA1c - Insulin resistance (↓ IRS-1, phosphorylation in response to insulin) - ↑ basal lipolysis - ↑ macrophage infiltration - ↑ adipocytes size - ↓ adiponectin
Adipose-devired stem cells (ADSCs)					
Rusin et al. (7)	2Gy 1.40Gy/min (LDR) 7.31Gy/min (HDR)	γ-rays (Cs^137^)	Human ADSCs	*In vitro*	- Cell cycle perturbation in G2/M accumulation (more pronounced inhibition with HDR) - ↓ p53
Haubner et al. (37)	6MV 2; 6; 12Gy 3Gy/min	X-rays	- Human dermal microvascular endothelial cells (HDMEC) - Human ADSCs from subcutaneous fat tissue	*In vitro*	- HDMEC: ↓ dose-dependent; ↑ IL-6, FGF, ICAM-1 - ADSCs (stem cell medium): proliferation ↓ at 6–12 Gy, ↑ IL-6 - ADSCs (endothelial medium): ↓ dose-dependent, ↑ FGF - HDMEC + ADSCs (co-culture): ↓ (less pronounced than HDMEC alone); ↑ IL-6, FGD, ICAM-1
Schröder et al. (38)	0.01; 0.05; 0.1; 0.3; 0.5; 1; 2Gy 0.54Gy/min	X-rays	Human ADSCs from reduction mammoplasties	*In vitro*	- ↑ proliferation and survival (below 0.1Gy) - ↓ proliferation and long-term survival (>0.1-2Gy) - ↑ of dose-dependent DNA damage - modest ↑ MMP-2 at 0.5 Gy
Hammad et al. (36)	125 kV, 4.7 mA 0; 0.5; 1; 2; 4Gy 2 Gy/min^2^ 95 MeV/nucleon LET ~ 33 keV/μ, 2Gy/min^3^ 110–125 MeV 2Gy/min^4^	^2^X-rays ^3^Carbon ions ^4^Protons	Human ADSCS (some groups pre-treated with Nrf2 inhibitor)	*In vitro*	- ↓ cell survival - Carbon ions most cytotoxic, followed by protons then X-rays - Nrf2 inhibition significantly sensitized ADSCs to radiation across all modalities with reduced LD50 - ↓ adipogenic and osteogenic differentiation - ↓ alkaline phosphatase
Taeb et al. (39)	5; 10; 30Gy 0.28 Gy/min	γ-rays (Co^60^)	Human ADSCs	*In vitro*	- ↓ viability (10 and 30Gy) - ↑ ABCA1 mRNA and MMP-9 protein expression (5Gy)
Jeong et al. (40)	6 MeV 18Gy	Electrons	Female pigs ADSCs isolated from subcutaneous adipose tissue	*In vivo* (irradiation)	- ↓ proliferation - ↑ senescence - Impaired adipogenic and altered chondrogenic differentiation
Du et al. (21)	Low LET:^2^ ~ 0.8Gy/min Hight LET:^3^ 80–100 MeV/u ~ 0.25Gy/min	^2^X-rays ^3 12^C^6+^ ions	ADSCs	*In vitro* (2D cultured cells and 3D sphere culture)	- 3D ADSCs ↑ radioresistant - 3D ADSCs ↑ differentiation potential - 3D ADSCs ↑ stem-like cell percentage - ↓ potential of transformation
Michaelidesová et al. (22)	MV 1; 2; 4; 8Gy	X-rays	- Human breast cancer cell line MCF7 - Human ADSCs	*In vitro*	- MCF7: ↑ radiosensitivity and apoptosis, ↓ proliferation - ADSCs: ↓ radiosensitivity and apoptosis
Baaße et al. (24)	0.25; 0.5; 2; 4; 6; 8Gy 3.75Gy/min	X-rays	- Human ADSCs isolated from reduction mammoplasties - Human breast cancer cell lines (MCF-7 and ZR-75-1) - Non-tumorigenic epithelial breast cell line (MCF10A)	*In vitro*	- ADSCs: intermediate radiosensivity, ↓ survival fraction, ↓ proliferation (≥ 2Gy), G0/G1 arrested, DNA damage checkpoint activation, ↑ p21 - MCF-7 / ZR-75-1: ↑ radiosensitive than ADSCs, strong proliferation inhibition - MCF10A: most resistant and minor effects on proliferation
Long-term metabolic effects on adipose tissue					
Kartal et al. (25)	Radiotherapy (46% of children had received radiotherapy)	n/s	Leukemia and lymphoma survivors at least 2 years after treatment aged ≥ 6	*In vivo*	- ↑ obesity
Wei et al. (45)	Total body irradiation 10–14.4Gy	n/s	Adult acute lymphoblastic leukemia survivors of childhood hematopoietic stem cell transplantation and total body irradiation	*In vivo*	- Abnormal body composition - ↑ prevalence of abnormal glucose tolerance - ↓ insulin sensitivity - ↓ adiponectin
Huang et al. (27)	Total body or abdominal irradiation in childhood	n/s	- Adult survivors of childhood cancer - Subcutaneous adipose biopsies from adult childhood cancer survivors	*In vivo*	- Activation of the TREM2-TYROBP - Macrophage expansion - ↓ adiponectin - ↑ PAI-1 - Altered leptin–adiponectin ratio - ↑ fasting glucose and HbA1c (in survivors with dysregulated adipokine profiles)
Friedman et al. (26)	Abdominal irradiation	n/s	Cancer survivors under 21 years	*In vivo*	- ↑ Risk for insulin resistance and diabetes
Yoshizumi et al. (46)	Full-thickness dorsal skin irradiation 40Gy in 4 fractions of 10Gy	X-rays	7- week-old male BALB/cfC3H mice	*In vivo*	- Radiodermatitis - ↑ ulceration (after 6 months) - ↓ tissue oxygen saturation - Subcutaneous fat atrophy - ↓ ADSCs - Impaired wound healing
Adipocytes interaction with tumor cells					
Bochet et al. (23)	130 kV 0,478Gy/min	X-rays	- -Murine 3 T3-F442A pre- adipocyte cell line (differentiated adipocytes status at irradiation) - SUM159PT human breast cancer cells	*In vitro*	- ↑ survival / radioresistance - ↑ effector kinase Chk1 - ↑ IL-6
Shreder et al. (41)	250 kV, 16 mA 0.5; 2; 10Gy 1Gy/min	X-rays	- Primary human subcutaneous pre-adipocytes - SGBA pre-adipocytes	*In vitro*	- ↓ proliferation and clonogenic survival - ↑ lipid accumulation (10Gy) - Pre-adipocytes radioresistant
Kosmacek et al. (49)	5Gy 2Gy/min	X-rays	- Adipocytes isolated from gonadal fat pads of C57BL/6 J mice - Fibroblasts isolated from prostates of C57BL/6 J mice Cancer cell lines (human): Prostate carcinoma (PC3) and colorectal carcinoma (HT29)	*In vitro*	- ↓ senescence and contractibility - Adipocytes: adiponectin secretion (↑ viability, ↓ senescence, ↓ activation). - Cancer cells: ↓ survival

γ, gamma radiation; n/s, not specified. The superscript numbers are used to establish the correspondence between each radiation protocol and the respective type of radiation.

### Oxidative stress and cellular damage

Radiation exposure disrupts adipose tissue homeostasis, providing free radicals that can induce lipid oxidation and other chemical changes (indirect effect), as oxidative stress generation via nicotinamide adenine dinucleotide phosphate (NADPH) oxidase pathway activation and impairment of antioxidant defense systems. Poglio et al. (9) demonstrate dose-dependent increases in reactive oxygen species production, with elevated NADPH oxidase expression and reduced antioxidant enzyme activity following radiation exposure, enhanced after 10 Gy irradiation when compared to 7 Gy, suggesting that the underlying cause of apoptotic cells was attributed to oxidative stress (9).

The radiation-induced oxidative inflammation and senescence imbalance trigger cellular damage, affecting both adipocytes and SVF cells, resulting in apoptotic cell events predominantly within the SVF (9, 33, 34). This imbalance is consistent with the findings by Ruggiero et al. (35), who demonstrated a persistent reduction and functional impairment of adipose progenitor cells following irradiation. These facts suggest that radiation preferentially targets proliferating progenitor populations over mature, metabolically active adipocytes (33, 35).

The study conducted by Hammad et al. (36) investigated the role of oxidative stress conditions on ADSCs after exposure to X-rays, protons, and carbon ion irradiation. A significant reduction in the survival of ADSCs was observed for all types of radiation, being further impaired when submitted to nuclear factor NF-E2-related factor 2 (Nrf2) inhibitor before irradiation: median lethal dose without Nrf2 was 3.5 for X-rays, 2.23 for protons and 1.61 for carbon ions; median lethal dose under Nrf2 inhibition was 2.33 for X-rays, 1.60 for protons and 1.24 for carbon ions. Moreover, adipogenesis was significantly reduced after X-ray irradiation and further reduced under oxidative stress-promoting conditions across all radiation types (36).

### Impaired adipogenesis, stem cell dysfunction, and inflammatory reprogramming

Ionizing radiation exposure severely compromises adipose tissue regenerative capacity by targeting ADSCs and preadipocytes. Multiple studies demonstrate reduced ADSCs proliferation, increased senescence, and impaired differentiation capacity following radiation exposure (7, 21, 23, 24, 27, 34, 35, 37–40).

The effects of ionizing radiation on adipose tissue usually focus on ADSCs, because they are mesenchymal multipotent stem cells responsible for recruiting immune cells, expressing cytokines and other molecules to promote repair, and may undergo differentiation to replenish depleted cell populations (7).

ADSCs might also compensate for endothelial dysfunction after irradiation by balancing the expression of soluble adhesion molecules (37). Interestingly, low-dose irradiation may further enhance such an effect. Schröder et al. (38) explored the proliferation-enhancing effect of X-rays low-dose irradiation (defined by the authors as ≤ 0.1 Gy) in ADSCs 48 h post-plated. The long-term survival of ADSCs remained unchanged, and no cytotoxic or genotoxic side effects were detectable. At 0.5 Gy, tissue repair mechanisms such as metalloproteinase 2 were upregulated (38). When in co-culture with endothelial cells, ADSCs exposed to X-rays (12 Gy) were also shown to release higher levels of basic growth factor, intercellular adhesion molecule-1 (ICAM), and interleukin-6 (IL-6) (37).

Ionizing radiation induces a range of lesions in genomic DNA, widely regarded as its primary subcellular target. The resulting DNA damage activates the p53 signaling pathway, leading to cell cycle arrest and cellular senescence (23, 35). The molecular basis involves cell cycle arrest mediated by p21 upregulation, DNA repair mechanism activation, and metabolic reprogramming. Regarding the harmful effects of radiation on ADSCs, Baaße et al. (24) investigated the cytotoxic effects and DNA repair after irradiation of ADSCs isolated from the breast. X-rays irradiation with 0 to 8 Gy (dose rate 3.75 Gy/min) did not change the immunophenotype of ADSCs. However, based on clonogenic survival fraction and cell proliferation assays, the number of colonies decreased with increasing irradiation dose, assigned as an intermediate radiation sensitivity, and ADSCs showed a lower proliferation rate, possibly due to apoptosis or cell cycle arrest, the latter being associated with the induction of DNA repair mechanisms mediated by p21. The knowledge of the extent of possible damages to ADSCs after beam exposure may be important for planning radiation treatment, because they are crucial for the healing of many tissue damages (24).

Another study (37) using unprocessed subcutaneous adipose tissue samples obtained from patients undergoing elective body-contouring procedures, irradiated with X-rays of 2 Gy, 6 Gy, and 12 Gy in a 6 MV energy linear accelerator, also demonstrated significantly reduced ADSCs number after irradiation with 6 and 12 Gy, but not after irradiation with 2 Gy (37). Using the total dose of 2 Gy, Rusin et al. (7) investigated the differences in human primary ADSCs’ response to two different dose rates (1.40 Gy/min and 7.31 Gy/min) from gamma irradiation, also showing minimal effects on cell proliferation. However, cells exposed to the high-rate dose experienced a decrease in the number of healthy cells. They also observed a lower number of cells in the G0/G1 and S cell cycle phases and a higher number of cells in the G2/M phase. Low-rate dose also caused an increase in cells in the G2/M phase, but with less pronounced effect. No significant upregulation of p53 was observed. Low-rate dose cells showed a higher number of senescent cells compared to high-rate dose ones; however, similar senescence levels in control were observed, suggesting that this response may be a normal behavior of ADSCs under the experimental conditions used (7). Taeb et al. (39) also evaluated the effects of different doses of gamma irradiation on the survival of ADSCs from breast tissue of a breast cancer patient with 5, 10 and 30 Gy (dose rate 0.28 Gy/min) on a Theratron Co60 therapy unit. Irradiation with 10 and 30 Gy gamma irradiation decreased ADSCs’ viability, while the 5 Gy increased ABCAI1 and MMP-9 protein expression (39).

The function and morphology of ADSCs have also been studied after whole-body irradiation. In a study performed in micro pigs (40), effects analyzed in ADSCs derived from subcutaneous adipose tissue in the irradiation area with a single dose of 18 Gy through a 6 megaelectron-volt (MeV) electron beam on a linear accelerator. Electron beam irradiation inhibited the proliferation and induced senescence of isolated ADSCs 6 weeks after irradiation. After irradiation, ADSCs lost the capacity for adipogenic and chondrogenic differentiation, but retained the capacity for osteogenic differentiation, which can contribute later to tissue fibrosis (40).

In the study by Du et al. (21), primary cultures of ADSCs were surgically obtained from subcutaneous adipose tissue of a human donor, and then a monolayer (2D) and sphere culture (3D) ADSCs cultures were irradiated with low LET X-rays (dose rate ~0.8Gy/min) and high LET carbon ion (^12^C^6+^) beams (80–100 MeV/u). After irradiation, 3D cultures demonstrated enhanced radioresistance compared to 2D cultures, suggesting microenvironmental factors influence radiation sensitivity. This is supported by the higher clonogenic survival fraction of 3D cultures, compared to 2D cultures. Moreover, high LET ^12^C^6+^ irradiation induced more cell death than low LET X-rays. 2D and 3D cultures were also alternatively irradiated continuously with a fraction dose of 2 Gy per day for 5 days. There was no transformation colony in the month after irradiation, suggesting that ADSCs had low tumorigenicity under the stress of ionizing irradiation. Thus, both samples have a lower potential for transformation and were suggested for use as evidence in the irradiation risk assessment and in understanding irradiation protection against side effects during radiotherapy. The authors proposed further studies to identify why the ADSCs are relatively genomically stable and resistant to genotoxic stress (21).

Ionizing irradiation, such as X-rays, induces profound inflammatory reprogramming within adipose tissue, characterized by macrophage infiltration and activation, enhanced pro-inflammatory cytokine production, and altered adipokine secretion profiles (27, 34). Reported alterations include decreased adiponectin (anti-inflammatory, insulin-sensitizing) and increased *PAI-1* (pro-thrombotic, pro-inflammatory) secretion. Gene expression analysis reveals upregulation of inflammatory markers, including *TREM2*, *TYROBP*, and *SPP1*, indicating sustained inflammatory activation. This inflammatory milieu contributes to insulin resistance development and metabolic dysfunction while potentially creating a pro-tumorigenic microenvironment (27, 34). These findings suggest a model of radiation-induced inflammatory reprogramming, sustained by cellular stress, immune-cell recruitment and inflammatory signaling, contributing to impaired adipose tissue homeostasis.

### Structural and functional remodeling

Radiation-induced structural alterations include reduced mature adipocyte number, increased average cell size, and impaired tissue expandability. These changes reflect compromised adipogenesis, reduced hyperplastic capacity, and altered lipolytic activity (6, 9, 35, 41–43).

Marques et al. (6) studied whether low-dose ionizing radiation could modulate the capacity of murine 3 T3-L1 pre-adipocytes to regulate angiogenic function after being exposed to X-rays at 0.1, 0.3, 0.5, 0.8, or 1 Gy (dose rate 300MU/min) using a linear accelerator X-rays photon beam, one day after differentiation induction. The authors observed a lower number of mature adipocytes derived from exposed pre-adipocytes, despite the low doses used (effects only at 0.3 Gy). Interestingly, adipocyte-conditioned media demonstrated enhanced pro-angiogenic properties, especially for the 0.3 Gy ionizing radiation (6). In turn, Shreder et al. (41) exposed adipocytes to single X-rays doses of 0.5, 2 and 10 Gy (dose rate 1 Gy/min) right after differentiation induction. They observed lower proliferation and clonogenic survival of human primary pre-adipocytes at 2 Gy and a completely inhibited proliferation at 10 Gy. Triglyceride content in adipocytes was enhanced at 10 Gy, but no significant alterations were found in the expression of PPARG or CEBPA, meaning that the differentiation potential of pre-adipocytes was mostly unaffected. The secretion of adiponectin, leptin and visfatin also remained unchanged (41). Another protocol in C57BL/6 mice (35), which were irradiated with a total dose of 7 Gy in two 3.5 Gy fractions (dose rate 0.358 Gy/min), with less than 10 min between exposures from a cobalt (Co60) source, showed that the progenitor cell differentiation and proliferation capacity were compromised in adipose tissue, due to the formation of a lower number, but larger adipocytes from preadipocytes. However, no differences in metabolic health and senescence were observed when assessed by p21 and 8-OHdG staining in epidydimal and mesenteric AT (35). In another study in mice, Ablamunits et al. (43) γ-irradiated ob/ob mice with total body irradiation of 8 Gy from a cesium (Cs137) source, followed by bone marrow transplantation. As a result, 2 months after irradiation, the fat mass gained was arrested. The authors reported a reduction in smaller adipocytes on SAT, which may suggest that preadipocyte proliferation and differentiation were affected in irradiated mice (43). In a similar study (44) also using ob/ob mice exposed to 8.5 Gy, a reduction of body weight gain was observed after 8–10 weeks. Also, although authors did not observe significant differences in the size or morphology of adipocytes, abdominal irradiation slightly decreased abdominal adipose tissue weight and caused macrophage infiltration (44).

In C57BL/6 mice irradiated with gamma radiation at 10 Gy and 7 Gy, given in a single administration (dose rate 4.4 Gy/min) (9), the SAT presented morphological changes, including reduced mature adipocyte size and increased number of small adipocytes at both 7 and 10 Gy. Accordingly, adipocyte differentiation was severely impaired in cultures obtained from irradiated animals compared to non-irradiated controls, also demonstrating lower triglyceride storage. More apoptotic cells were identified in animals irradiated with lethal (10 Gy) and sub-lethal (7 Gy) doses, but most of these cells were from the SVF (9).

A study (42) in non-human primates irradiated with a total dose of 4 Gy (dose rate 0.8 Gy/min) on a clinical linear accelerator with 6 megavolts (MV) energy demonstrated larger adipocyte cell size in the subcutaneous adipose tissue. X-rays irradiated animals with a total dose of 4Gy also showed lower insulin receptor substrate 1 (IRS1) activation in response to insulin, insulin resistance, and higher density of macrophages, negatively associated with adiponectin levels, and transforming growth factor-beta signaling. As a result, animals had decreased capacity for SAT expansion through differentiation and hyperplasia, resulting in type 2 diabetes (42). A similar study (35) showed decreased proliferative capacity in VAT adipocytes, as well as impaired differentiation and expansion when cultured (35). Furthermore, CD34 staining was decreased in the adipose tissue, suggesting impaired progenitor differentiation ability. No senescence was found in the adipose tissue or liver of irradiated animals when evaluated through β-galactosidase, 8-OHdG, p21 and p16 (35).

## Radiation-induced long-term metabolic impairment in cancer survivors

Patients undergoing body irradiation as radiotherapy may have effects that can modify adipose tissue plasticity and reconstructive capacity, which may also contribute to metabolic diseases later in life (9, 25–27). After total body gamma irradiation (7 Gy or 10 Gy), Poglio et al. (9) observed changes in the morphologic and metabolic characteristics of subcutaneous adipose tissue in mice (9). Children diagnosed with leukemia and lymphoma who survived at least 2 years after completing all treatments (46% of children had received radiotherapy) demonstrate significantly higher obesity and metabolic syndrome prevalence, features consistent with insulin resistance (25). Wei et al. (45) studied young adult acute lymphoblastic leukemia survivors who underwent childhood hematopoietic stem cell transplantation and total body irradiation, showing a higher prevalence of abnormal glucose tolerance and lower insulin sensitivity and adiponectinemia than groups not exposed to ionizing irradiation, suggesting long-term metabolic dysfunction (45). Huang et al. (27) studied the subcutaneous adipose tissue biopsies from childhood cancer survivors who received abdominal radiotherapy or total body irradiation to obtain tissue for bulk RNA sequencing. Gene expression indicated adipose tissue dysfunction, namely macrophage infiltration, inflammation (upregulation of *TREM2*, *TYROBP* and *SPP1*) and dysregulated adipokine secretion, marked by decreased adiponectin and increased *PAI-1* (27). Another study from Friedman et al. (26) reveals that cancer survivors under 21 years exposed to abdominal radiation face increased risk for insulin resistance and diabetes without evidence of beta-cell dysfunction, suggesting adipose tissue involvement rather than pancreatic damage (26).

Moreover, evidence suggests that long-term metabolic changes in adipose tissue can be related to persistent alterations in the ADSCs, which are responsible for regeneration. After whole-body irradiation, Jeong et al. (40) showed that electron beam irradiation reduced the proliferation, increased senescence, and reduced adipogenic differentiation of isolated ADSCs (40). Another radiation-induced long-term analysis (46) showed a significant reduction of ADSCs after irradiation, suggesting reduced wound healing capacity, which can be associated with fibrosis, fat atrophy, ischaemia, and impaired wound healing, especially after 6 months (46). However, the contribution of persistent radiation-induced cellular senescence to long-term metabolic changes in adipose tissue remains uncertain due to the few studies addressing long-term effects *in vivo*. Radiation is one of the factors that may induce ADSCs senescence, and senescence may impair future adipose tissue plasticity and adaptation to metabolic fluxes, as reviewed by Foti et al. (47). However, studies addressing the causality between ionizing radiation, ADSCs senescence and long-term metabolic defects are lacking. Liermann-Wooldrik et al. (34) investigation showed higher adipocyte senescence after ionizing radiation, remaining elevated after 6 months, which can be related to reduced adipogenesis, impaired adipose function, and potentially to maintaining tissue integrity upon DNA damage (34). However, Ruggerio et al. (35) observed no differences in markers of cellular senescence, despite adipose tissue impairment (35). Although senescence is characterized by cell-cycle arrest, the chronic state of radiation-induced senescent cells can produce a senescence-associated secretory phenotype (SASP) that may contribute to pro-inflammatory factor secretion and many late tissue damage (48).

## Adipose tissue-tumor cells interactions in the radiation context

Cancer is a disease in which malignant cells interact dynamically with multiple normal cell types such as fibroblasts, adipocytes, infiltrating immune cells and endothelial cells within the context of the extracellular matrix. Despite being among the most abundant cell types within the tissue microenvironment, particularly in the breast, adipocytes remain one of the least well-studied cell types in the context of ionizing radiation (23).

Given the possible biological effects of ionizing radiation on adipose tissue, understanding how the changes occurring in the adipose tissue microenvironment shape tumor behavior and response to irradiation is of utmost importance (7, 9, 21, 23, 24). The close anatomical relationship between adipose tissue and tumor cells in many solid malignancies creates complex interactions that influence treatment outcomes. In pathological conditions, adipocytes secrete significant amounts of adipokines and other pro-inflammatory factors, which have been shown to promote tumor growth and expansion and are associated with angiogenesis, which plays a critical role in tumor expansion. On the other hand, invasive cells dramatically impact surrounding adipocytes, changing their phenotype and specific biological features that reshape the tumor microenvironment (23, 41).

Using primary adipocytes from gonadal fat pads and fibroblasts from the prostate of mice after X-rays exposure to 5 Gy of radiation, Kosmacek et al. (49) demonstrated that adipocytes and exogenous adiponectin protect primary fibroblasts from radiation-induced cell death, myofibroblast transformation and cell senescence. Fibroblasts alone had reduced cell viability, while adipocyte viability was unchanged. Since adipocytes secrete a broad range of adipokines, the authors inactivated adipocytes’ conditioned media through heat and the protection was ablated (49).

Considering the lack of knowledge about the impact of irradiation on pre-adipocytes and adipocytes, both residing in fat tissue close to tumors and inflamed joints, the radiation response of human primary pre-adipocytes concerning the release of adipokines, survival and differentiation potential of pre-adipocytes, an *in vitro* study was developed to investigate the radiation effects of the adipocyte and tumor cells crosstalk on breast cancer cell after irradiation. For that, pre-adipocytes differentiated into mature adipocytes were co-cultured with breast cancer cells and then submitted to a 130 kilovolt (kV) X-rays machine (dose rate 0.478 Gy/min). Co-culture with adipocytes significantly decreased cytotoxicity in tumor cells after irradiation. Post-mitotic dying cells showed abnormal mitosis and the formation of large cells with multiple micronuclei. Based on the phosphorylation pattern of the two effector kinases Chk1 and Chk2, adipocytes protected tumor cells from ionizing radiation-induced toxicity, preventing mitotic cell death through the induction of molecular factors such as Chk1. These findings suggest that adipocytes located in the tumor microenvironment promote radioresistance by inducing IL-6 and activating Chk1 (23). Michaelidesová et al. (22) irradiated human ADSCs and MCF-7 breast cancer cells with megavoltage X-ray doses of 1, 2, 4 and 8 Gy. The outcomes showed that ADSCs were less sensitive to radiation than breast cancer cells, as indicated by a higher ADSCs survival ratio and lower ADSCs levels of radiation-induced apoptosis (22). In summary, the presence of adipocytes in co-culture with tumoral cells apparently protects them from ionizing radiation. However, given the inconsistent data regarding adipokine secretion, the mechanisms involved are still unknown. Such effects may involve other adipose tissue cells since ADSCs have been shown to be particularly resistant to irradiation.

## Conclusions and future perspectives

Despite the different protocols used in the available studies, most of the studies on adipocytes showed alterations in adipocyte size, a decrease in mature adipocytes, lower differentiation, alterations in senescence, and higher macrophage density, leading to arrested body weight in animal models. Moreover, the studies on ADSCs highlighted the lower proliferation, cell survival, viability, and the higher apoptosis rate. Thus, adipose tissue submitted to ionizing radiation tends to present markers of dysfunction, although the mechanisms may be different according to the many different irradiation approaches (high or low dose, dose rate) and different times after irradiation. The dynamic interplay between adipocytes and tumor cells shapes a microenvironment that can influence tumor behavior (tumor growth) and the therapeutic outcome (radioresistance). Thus, adipose tissue dysfunction caused by irradiation may not only lead to future metabolic complications but also influence the tumor microenvironment and the therapeutic response given the close relation between adipose and tumor cells.

Understanding the effects of ionizing radiation on adipose tissue is increasingly important given its rising use for diagnostic and therapeutic procedures. This evidence and the multiple biologically relevant metabolic functions of adipose tissue highlight the need for further studies, underscoring research into white and brown adipose tissue from multiple anatomic depots, as well as into the underlying browning mechanisms of adipose tissue. An important limitation of this review is the very limited number of studies addressing long-term effects of ionizing radiation in adipose tissue. This topic is of utmost relevance, and future studies should focus on elucidating such effects. Also, new studies with ionizing radiation approaches in the medical field should be conducted to enable a precision analysis of the impaired radiation-induced adipose tissue function, tailored to each person, taking into account the type of radiation and dose protocol.
